# A case report of a midesophageal diverticulum mimicking a fibrovascular esophageal polyp

**DOI:** 10.1016/j.ijscr.2019.05.047

**Published:** 2019-05-31

**Authors:** Kyle G. Mitchell, Erin M. Corsini, Robert M. Van Haren, Garrett L. Walsh, Boris Sepesi

**Affiliations:** a Department of Thoracic and Cardiovascular Surgery, University of Texas MD Anderson Cancer Center, Houston, TX, United States; b Department of Surgery, University of Cincinnati Medical Center, Cincinnati, OH, United States

**Keywords:** Case report, Esophageal diverticulum, Fibrovascular polyp, Esophageal diseases

## Abstract

**Introduction:**

Esophageal diverticula and esophageal fibrovascular polyps are uncommon clinical entities. While an asymptomatic presentation is possible, symptoms, when present, may be dissimilar in their gastrointestinal or respiratory characteristics. Additionally, these findings typically occur in different segments of the esophagus, with polyps occurring most frequently in the cervical esophagus and the midesophagus being the predominant location of pathologic diverticula.

**Presentation of case:**

We report the case of a 55-year-old patient who presented with a two-year history of progressive dysphagia secondary to a large proximal to midesophageal mass. Workup included esophagography, computed tomography, and endoscopy with ultrasound and was initially consistent with a diagnosis of a large esophageal fibrovascular polyp. Upon operative exploration, the mass was found to be a midesophageal diverticulum associated with a leading lipoma. The patient was successfully treated with transthoracic stapled diverticulectomy. At postoperative follow-up the patient was tolerating oral intake with no symptoms of dysphagia.

**Discussion:**

Esophageal diverticula are typically found in the midesophagus and are thought to arise from radial traction secondary to mediastinal inflammation. Esophageal fibrovascular polyps may result from tracheobronchial compression, and esophagography typically identifies a mobile intraluminal mass.

**Conclusion:**

Esophageal fibrovascular polyps and diverticula are rare, and a high index of suspicion is important in evaluation of these entities.

## Highlights

• Esophageal diverticula and esophageal fibrovascular polyps are uncommon entities.
• These anomalies often present with different symptomatology and may be associated with specific esophageal anatomy.
• We present a case of a midesophageal mass, which was suggestive of a fibrovascular polyp upon diagnostic workup.
• Operative exploration revealed the mass to be an esophageal diverticulum with a leading lipoma.
• Esophageal diverticula may arise in the midesophagus secondary to inflammation or traction, such as a leading lipoma.

## 1 Introduction

Midesophageal diverticula are frequently asymptomatic and diagnosed incidentally. Unlike distal esophageal diverticula which are often associated with gastroesophageal reflux disease, they are classically associated with chronic mediastinal inflammation and result from traction forces on the esophageal wall [[1], [2], [3], [4]]. However, it is not unusual for midesophageal diverticula to be asymptomatic and discovered incidentally [5]. Fibrovascular polyps (FVP) of the esophagus are similarly rare. They often arise below the cricopharyngeus and may extend in the submucosal plane to the stomach [6,7].

We report the case of a 55-year-old woman whose workup supported the diagnosis of a large FVP. On exploration, the lesion was found to be a midesophageal diverticulum traveling in the submuscular plane. The lesion was successfully managed with transthoracic diverticulectomy and buttressed closure.

This work has been reported in line with the SCARE criteria [8].

## 2 Case presentation

**Fig. 1:**
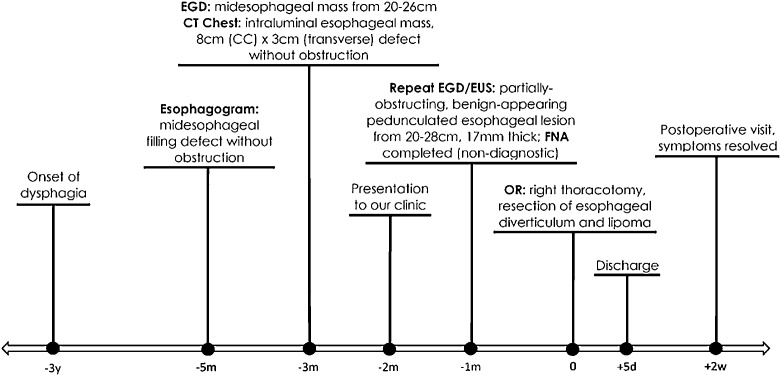
**Timeline of symptoms, diagnostic testing, intervention, and postoperative course**. EGD = esophagogastroduodenoscopy, CT = computed tomography, EUS = endoscopic ultrasound, FNA = fine needle aspiration, OR = operating room, m = months, w = weeks, d = days.

A 55-year-old healthy woman was referred to our institution with a two-year history of progressive dysphagia to solids (Fig. 1). She reported a recent episode of solid food getting stuck in her throat, which prompted presentation to an outside endoscopist. The patient reported no alcohol use. She was a former smoker with a 15 pack-year history, but had quit over 20 years prior. The patient had a past medical history of gastroesophageal reflux disease, for which she was taking omeprazole, and hypothyroidism. She had no known history of any esophageal dysmotility disorder. There was a history of diabetes mellitus in her mother and son.

**Fig. 2:**
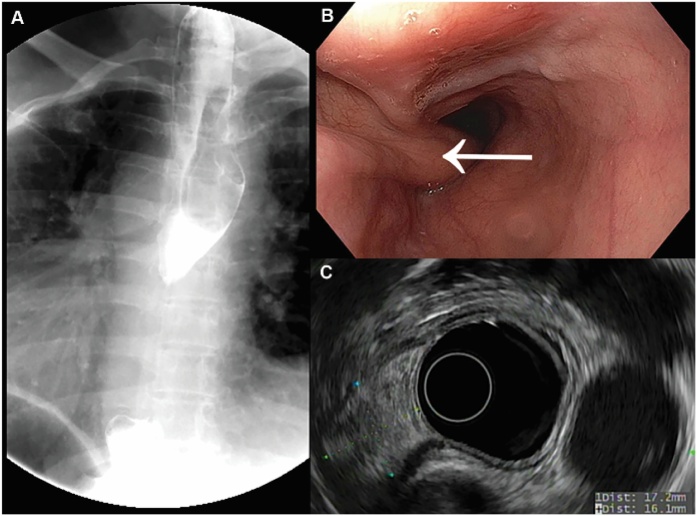
**Diagnostic evaluations of a patient presenting with a two-year history of dysphagia**. (A) Esophagogram demonstrating an intraluminal mass; (B) Endoscopic view of pedunculated lesion (*arrow*); (C) EUS demonstrating apparent submucosal origin.

Physical exam and laboratory testing were unremarkable. Esophagography demonstrated a filling defect in the upper thoracic esophagus. Computed tomography (CT) demonstrated an 8 cm mass. Endoscopic ultrasound (EUS) demonstrated a pedunculated mass with a submucosal origin beginning at 20 cm from the incisors on the right side of the neck (Fig. 2). The lesion was felt to have the characteristic appearance of a FVP and the patient elected to proceed with resection.

**Fig. 3:**
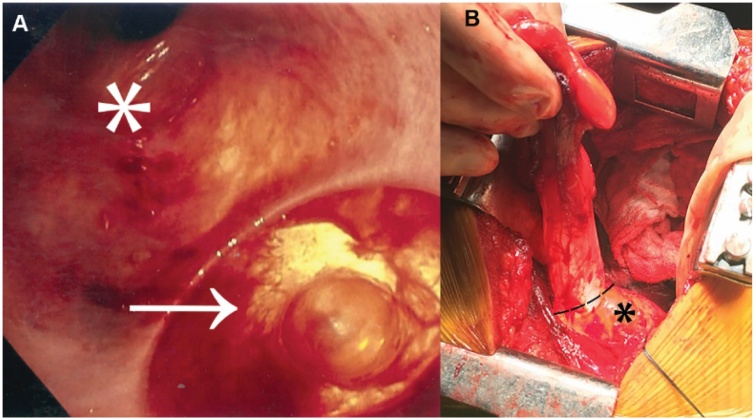
**Intraoperative findings during planned resection of suspected esophageal mass**. (A) Esophagoscopy after mobilization of the diverticulum revealed a patent esophageal lumen (*asterisk*) and diverticular ostium (*arrow*); (B) Intraoperative photograph; esophagus (*asterisk*) and approximate level of resection (*dashed line*) are marked.

The exploration began via a right cervical approach. The recurrent laryngeal nerve was identified and the cervical esophagus was mobilized. The mass was palpable on the posterior esophageal wall at the thoracic inlet. Upon a short myotomy, no stalk was identified and the mass could not be delivered to the neck. The cervical incision was closed and a right thoracotomy was performed. The mass was seen extending from the level of the azygos vein to the thoracic inlet. The esophageal muscular layer was intact. Following myotomy, the soft mass, which was densely adhered to the mucosa, was visualized and dissected from the underlying mucosa. It became evident that the mass maintained its attachment to a portion of the mucosa. Complete mobilization revealed the mass to be a lipoma at the tip of a large midesophageal diverticulum traveling in a submucosal plane. Repeat endoscopy demonstrated an ostium in the esophageal wall opening into a blind-ending pouch. The diverticulum was fully mobilized and resected using a stapler (Fig. 3). Mucosal closure was reinforced with overlying muscle and a pleural flap.

The patient was diagnosed with a large midesophageal diverticulum with a lead point lipoma. The patient’s postoperative course was uncomplicated. A postoperative esophagogram demonstrated no esophageal leak or obstruction. Pathology demonstrated a 7.5 cm diverticulum with a 4.5 cm lipoma without malignancy. At follow-up on the nineteenth postoperative day, the patient was tolerating a diet without dysphagia.

## 3 Discussion

Fibrovascular polyps classically present as pedunculated masses arising immediately distal to the cricopharyngeus [6,7,9]. Respiratory symptoms can result from tracheobronchial compression or regurgitation and airway occlusion [6]. Esophagography demonstrates an intraluminal mass in the cervical or upper thoracic esophagus. Mobility of the polyp and the normal appearance of its epithelial lining can make endoscopic diagnosis difficult [6,7]. Excision of the polyp is usually performed via a transcervical approach, though endoscopic and transthoracic approaches have been described [6,7,9,10].

Midesophageal diverticula are found near the carina and classically have been attributed to radial traction from mediastinal inflammatory processes [1,2]. When present, symptoms commonly include dysphagia and regurgitation, however an asymptomatic presentation is not unusual [1,5]. Treatment with diverticulectomy via open transthoracic or thoracoscopic approach is curative but can be associated with a substantial complication rate [1]. Transhiatal and uniportal thoracoscopic resection have been described [1,2,11].

This case is unique in its presentation in that workup supported a diagnosis of FVP. The intramural tract of the diverticulum mimicked the pedunculated stalk of a FVP. The diverticular ostium was collapsed and not identified on endoscopy until after full operative mobilization of the diverticulum. Unlike most reported cases of midesophageal diverticula, this was neither associated with mediastinal inflammation nor with an underlying motility disorder. The diverticular ostium was collapsed and not identified on endoscopy until after full mobilization of the diverticulum. The presence of a leading lipoma is also uncharacteristic. These findings highlight the importance of maintaining a high index of suspicion when evaluating a suspected FVP or midesophageal diverticulum, as well as astute use and interpretation of diagnostic imaging modalities in patient evaluation.

## Conflicts of interest

The authors have no conflicts of interest to disclose.

## Sources of funding

This research did not receive any specific grant from funding agencies in the public, commercial, or not-for-profit sectors.

## Ethical approval

MD Anderson Cancer Center Institutional Review Board – This investigation is exempt from ethical approval at our institution.

## Consent

Consent obtained.

## Author’s contribution

BS and GW contributed to conceptualization, study design, and manuscript drafting and editing. KM, EC, and RV contributed to data collection, data analysis, and manuscript drafting and editing.

## Registration of research studies

NA.

## Guarantor

Erin M. Corsini.

Boris Sepesi.

## Provenance and peer review

Not commissioned, externally peer-reviewed.
